# A global atlas of soil viruses reveals unexplored biodiversity and potential biogeochemical impacts

**DOI:** 10.1038/s41564-024-01686-x

**Published:** 2024-06-20

**Authors:** Emily B. Graham, Antonio Pedro Camargo, Ruonan Wu, Russell Y. Neches, Matt Nolan, David Paez-Espino, Nikos C. Kyrpides, Janet K. Jansson, Jason E. McDermott, Kirsten S. Hofmockel, the Soil Virosphere Consortium, Jeffrey L. Blanchard, Xiao Jun A. Liu, Jorge L. Mazza Rodrigues, Zachary B. Freedman, Petr Baldrian, Martina Stursova, Kristen M. DeAngelis, Sungeun Lee, Filipa Godoy-Vitorino, Yun Kit Yeoh, Hinsby Cadillo-Quiroz, Susannah G. Tringe, Archana Chauhan, Don A. Cowan, Marc W. Van Goethem, Tanja Woyke, Nicholas C. Dove, Konstantinos T. Konstantinidis, Thomas E. Juenger, Stephen C. Hart, David D. Myrold, Tullis C. Onstott, Brendan J. M. Bohannan, Marty R. Schmer, Nathan A. Palmer, Klaus Nüsslein, Thulani P. Makhalanyane, Katherine A. Dynarski, Neslihan Taş, Graeme W. Nicol, Christina Hazard, Erin D. Scully, Kunal R. Jain, Datta Madamwar, Andrew Bissett, Philippe Constant, Rafael S. Oliveira, Cristina Takacs-Vesbach, Melissa A. Cregger, Alyssa A. Carrell, Dawn M. Klingeman, Nicole Pietrasiak

**Affiliations:** 1 Biological Sciences Division, Pacific Northwest National Laboratory, Richland, WA USA; 2 School of Biological Sciences, Washington State University, Pullman, WA USA; 3 US Department of Energy Joint Genome Institute, Lawrence Berkeley National Laboratory, Berkeley, CA USA; 4 Institute for Chemical Research, Kyoto University, Kyoto, Japan; 5 Department of Molecular Microbiology and Immunology, Oregon Health & Science University, Portland, OR USA; 6 Department of Agronomy, Iowa State University, Ames, IA USA; 7 Biology, University of Massachusetts Amherst, Leverett, MA USA; 8 Institute for Environmental Genomics, University of Oklahoma, Norman, OK USA; 9 Department of Land, Air and Water Resources, University of California, Davis, Davis, CA USA; 10 Department of Soil Science, University of Wisconsin–Madison, Madison, WI USA; 11 Laboratory of Environmental Microbiology, Institute of Microbiology of the Czech Academy of Sciences, Prague, Czech Republic; 12 Microbiology Department, University of Massachusetts, Amherst, Amherst, MA USA; 13 Laboratoire Ampère, Ecole Centrale de Lyon, Ecully, France; 14 Department of Microbiology and Medical Zoology, Medical Sciences Campus, University of Puerto Rico, School of Medicine, San Juan, PR USA; 15 Australian Institute of Marine Science, Townsville, Queensland Australia; 16 School of Life Sciences, Arizona State University, Tempe, AZ USA; 17 Environmental Genomics and Systems Biology, Lawrence Berkeley National Laboratory, Berkeley, CA USA; 18 Molecular Biology Laboratory, Department of Zoology, Panjab University, Chandigarh, India; 19 Centre for Microbial Ecology and Genomics, Department of Biochemistry, Genetics and Microbiology, University of Pretoria, Pretoria, South Africa; 20 University of California, Merced, Merced, CA USA; 21 School of Civil and Environmental Engineering, and School of Biological Sciences, Georgia Institute of Technology, Atlanta, GA USA; 22 Department of Integrative Biology, University of Texas, Austin, TX USA; 23 Department of Life and Environmental Sciences and the Sierra Nevada Research Institute, University of California, Merced, Merced, CA USA; 24 Department of Crop and Soil Science, Oregon State University, Corvallis, OR USA; 25 Department of Geosciences, Princeton University, Princeton, NJ USA; 26 Institute of Ecology and Evolution, University of Oregon, Eugene, OR USA; 27 United States Department of Agriculture, Agricultural Research Service, Lincoln, NE USA; 28 Wheat, Sorghum and Forage Research Unit, Agricultural Research Service, United States Department of Agriculture, Lincoln, NE USA; 29 Department of Microbiology, Faculty of Science, Stellenbosch University, Stellenbosch, South Africa; 30 Climate and Ecosystem Science Division, Lawrence Berkeley National Laboratory, Berkeley, CA USA; 31 USDA-ARS Center for Grain and Animal Health Research Manhattan, Manhattan, KS USA; 32 Environmental Genomics and Proteomics Lab, Department of Biosciences, Satellite Campus, Sardar Patel University, Bakrol (Anand), India; 33 P. D. Patel Institute of Applied Sciences, Charotar University of Science and Technology, Changa, India; 34 Commonwealth Scientific and Industrial Research Organisation, Hobart, Tasmania Australia; 35 Centre Armand-Frappier Santè Biotechnologie, Institut national de la recherche scientifique, Laval, Quèbec Canada; 36 Department of Plant Biology, University of Campinas, Campinas, Brazil; 37 Department of Biology, University of New Mexico, Albuquerque, NM USA; 38 Biosciences Division, Oak Ridge National Laboratory, Oak Ridge, TN USA; 39 School of Life Sciences, University of Nevada Las Vegas, Las Vegas, NV USA

**Keywords:** Biogeography, Soil microbiology, Biogeochemistry, Microbial ecology

## Abstract

Historically neglected by microbial ecologists, soil viruses are now thought to be critical to global biogeochemical cycles. However, our understanding of their global distribution, activities and interactions with the soil microbiome remains limited. Here we present the Global Soil Virus Atlas, a comprehensive dataset compiled from 2,953 previously sequenced soil metagenomes and composed of 616,935 uncultivated viral genomes and 38,508 unique viral operational taxonomic units. Rarefaction curves from the Global Soil Virus Atlas indicate that most soil viral diversity remains unexplored, further underscored by high spatial turnover and low rates of shared viral operational taxonomic units across samples. By examining genes associated with biogeochemical functions, we also demonstrate the viral potential to impact soil carbon and nutrient cycling. This study represents an extensive characterization of soil viral diversity and provides a foundation for developing testable hypotheses regarding the role of the virosphere in the soil microbiome and global biogeochemistry.

## Abstract (web summary)

This study presents an extensive global compendium of metagenomically derived sequences that will serve as a foundation for understanding the role of viruses in soil ecosystems.

## Main

Viral contributions to soil ecology are largely unknown due to the extreme diversity of the soil virosphere. Despite variation in estimates of soil viral abundances (10^7^ to 10^10^ viruses per gram of soil), it is clear that soils are among the largest viral reservoirs on Earth^1–3^. Early metagenomics investigations have revealed high genetic diversity in soil viruses, with putative impacts on global biogeochemistry^1,2,4,5^. Still, less than 1% of publicly available viral metagenomic sequences are from soil^6^, reflecting the lack of knowledge about soil viruses and their ecological roles^4,7^.

High soil viral diversity may be due to the structural and/or physico-chemical heterogeneity of soils compared with other ecosystems^1,8–10^, as well as the high diversity of soil microbial hosts. Indeed, viral abundance and composition vary with factors such as soil pH, temperature, moisture, chemistry and habitat^10–12^. Much of this viral diversity is contained within DNA viruses, though RNA viruses also have the potential to influence soil processes^13,14^. While less is known about soil viral activity, a recent study of peatlands reported that close to 60% of soil viral genomes may be involved in active infections^15^, consistent with high activity observed in marine and other systems^4,16–18^.

Whether common macroecological patterns apply to the soil virosphere remains an open question; initial studies of the soil virosphere indicate that the ecology of viruses is at least partially decoupled from other microorganisms^8,10,19^. A major finding is that soil viral community turnover may occur over shorter spatial and temporal scales than microbial communities^8,10,19^. For instance, spatial viral turnover has been shown to be over five times higher than microbial community turnover across an 18 m soil transect^8^, and only 4% of peatland ‘viral operational taxonomic units’ (vOTUs) are shared across continents^20^. Other studies note the possibility for long-distance soil viral dispersal through atmospheric^21^ or aquatic transport^22^ consistent with low turnover. These contrasting results indicate a lack of consensus surrounding the spatial and temporal patterns of soil viruses and the need for large-scale surveys of the soil virosphere.

Importantly, soil viruses can influence biogeochemical cycling, antibiotic resistance and other critical soil functions by releasing carbon and nutrients during host infection and/or by altering host metabolism via auxiliary metabolic genes (AMGs)^9,15,18,23–28^. While soil AMG characterization is nascent^14^, marine systems demonstrate the breadth of functions ripe for discovery in soil^24^. More than 200 viral AMGs encoding functions related to carbon and nutrient cycling, stress tolerance, toxin resistance and other processes have been detected in marine systems^24^. In contrast, only a handful of these functions have been identified as soil viral AMGs^12,14,15,22,29,30^. AMGs encoding carbohydrate metabolism in particular may be present in soils, including a few that have been experimentally validated^9,10,15,29–31^.

Accordingly, understanding the role of viruses in soil ecosystems is one of the most pressing current challenges in microbial ecology^32^. Despite the expansion of studies characterizing soil viruses^4,12,29,30^, a comprehensive description of the global soil virosphere has yet to be performed. Such a description is necessary to begin to address questions regarding the spatiotemporal dynamics, physico-chemical interactions, host organisms and food web implications of the soil virosphere. In this Resource, we present a meticulous compilation of the Global Soil Virus (GSV) Atlas based on previous metagenomic investigations of worldwide soils. This atlas represents the most extensive collection of soil viral metagenomes so far, encompassing contributions from prominent repositories, ecological networks and individual collaborators.

## Results

### GSV Atlas

**Fig. 1:**
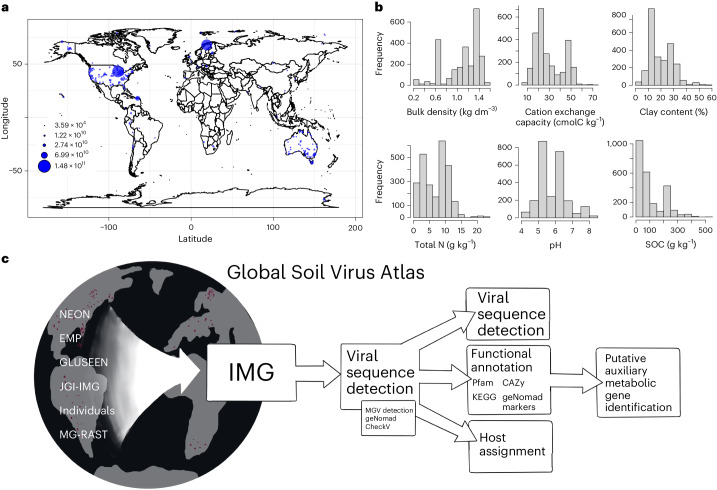
Data collection and workflow. **a**, The global distribution of samples, scaled by assembled base pairs. **b**, In order horizontally, histograms of mean soil bulk density (kg dm^−^^3^), CEC (cmol_c_ kg^−1)^, clay content (%), total nitrogen content (g kg^−1^), pH and SOC (g kg^−1^) associated with our samples from the SoilGrids250 database (0–5 cm). **c**, The sequence processing workflow.

**Fig. 2:**
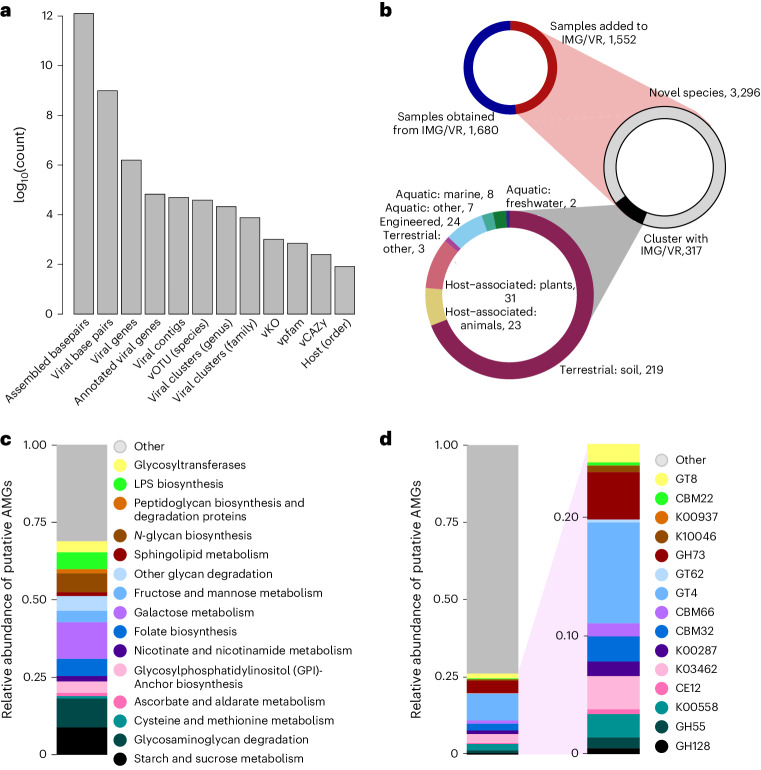
Data description. **a**, The count of each category across the full dataset. **b**, Top left: the proportion of samples obtained from IMG/VR versus the number of new samples contributed. Middle: within new samples, we identified 3,613 vOTUs of which 317 clustered with sequences already in IMG/VR. Bottom left: sequences in IMG/VR that clustered with vOTUs containing new sequences were mostly associated with soil habitats. **c**,**d**, The relative abundance of putative AMGs grouped by KEGG pathway (**c**) and by annotation (**d**).

For a description of the files contained by the GSV Atlas, please see ‘Data availability’ section. We amassed 1.25 × 10^12^ of assembled base pairs (bp) across 2,953 soil samples, including 1,552 samples that were not previously available in the US Department of Energy (DOE), Joint Genome Institute (JGI) Integrated Microbial Genomes and Microbiomes (IMG/M) database (Figs. 1 and 2). These samples were screened for viruses, yielding 616,935 uncultivated virus genomes (UViGs) of which 49,649 were of sufficiently high quality for further investigation (Methods). To quantify the extent of new viral diversity encompassed by the GSV Atlas, we compared sequences from samples not already in IMG/VR with those that were previously deposited. Newly contributed sequences clustered into 3,613 vOTUs of which only 317 clustered with existing viral sequences in IMG/VR. The vast majority associations with IMG/VR were with sequences previously uncovered from soil habitats (Fig. 2b).

We also collected associated environmental parameters describing each sample from the SoilGrids250m database^33^. We assayed a wide variety of soils that ranged from bulk density of 0.24–1.56 kg dm^−^^3^, cation exchange capacity (CEC) of 6.8–71 cmol_c_ kg^−1^, nitrogen content of 0.19–22.4 g kg^−1^, pH of 4.3–8.5, soil organic C (SOC) of 1.9–510.9 g kg^−1^ and clay content of 2.7–57.1% (Fig. 1).

The 49,649 UViGs of sufficient quality for downstream analysis clustered into 38,508 vOTUs at the species-like level^34^, of which 3,296 were previously unrepresented in IMG/VR (Fig. 2a,b). Only 13.9% of the GSV Atlas’ vOTUs appeared in more than one sample, and less than 1% were present in more than five samples. At higher taxonomic levels, we found 21,160 and 7,598 clusters at the genus and family levels, respectively^35^. This equates to an average of 40.01 (range 1–2,124), 35.48 (range 1–1,651) and 24.91 (range 1–896) unique viral clusters per sample at the species, genus and family levels. A total of 38,278 out of 38,508 vOTUs (99.4%) had at least one member assigned to a taxon by geNomad.

We identified 1,432,147 viral genes, of which only 260,258 (~18%) were found in at least one annotation database (1,022, 3,634 and 145 unique KO, Pfam and CAZy annotations, Fig. 2). After filtering to putative AMGs (Methods)^30^, we found 5,043 genes that mapped to 83 Kyoto Encyclopedia of Genes and Genomes (KEGG) pathways (1,941 KO and 3,357 CAZyme, some genes were associated with both KO and CAZyme). The median per sample putative AMG abundance (gene copies per sample) was ~19 genes (median 4, reflecting skewing from a few samples with high AMG abundance).

Some KEGG pathways represented by the most putative AMGs were associated with major soil carbon cycling processes (galactose metabolism and starch and sucrose metabolism). Likewise, at the level of gene annotations, the most common putative AMGs suggested a role for viruses in soil carbon cycles; including CAZymes like glycosyltransferase 4 (GT4), glycosylhydrolase 73 (GH73) and carbohydrate-binding module 50 (CBM50). Other abundant KEGG pathways and gene annotations (Fig. 2c,d) included glycosaminoglycan degradation (map00531), *N*-glycan biosynthesis (map00510), folate biosynthesis (map00790), 6-pyruvoyltetrahydropterin/6-carboxytetrahydropterin synthase (K01737) and 7-carboxy-7-deazaguanine synthase (K10026).

**Extended Data Fig. 1:**
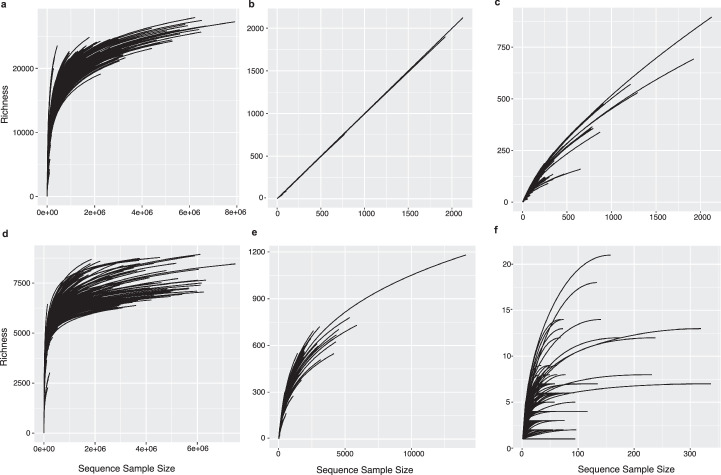
Rarefaction curves. **a**, Taxonomy from whole metagenomic sequences, **b**, vOTUs at the species level, **c**, viral clusters at the family level, **d**, Pfams from whole metagenomic sequences, **e**, Pfams from UViGs, **f**, CRISPR spacer-based host assignment of UViGs.

**Extended Data Fig. 2:**
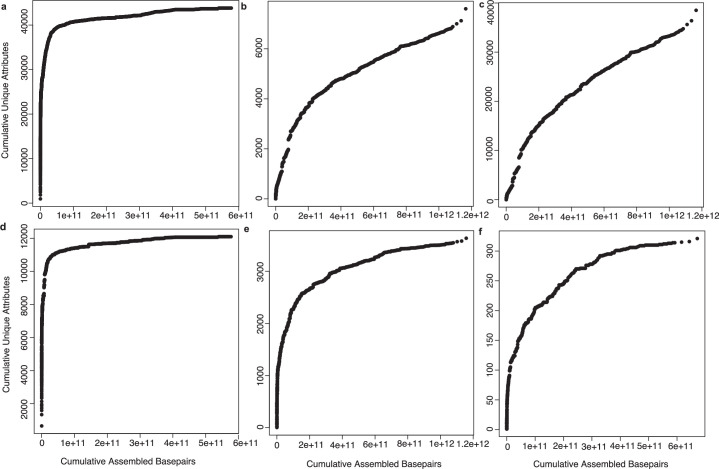
Total sequencing depth versus cumulative unique attributes. **a**, Taxonomy from whole metagenomic sequences, **b**, vOTUs at the species level, **c**, viral clusters at the family level, **d**, Pfams from whole metagenomic sequences, **e**, Pfams from UViGs, **f**, CRISPR spacer-based host assignment of UViGs.

Finally, in contrast to the saturation observed in rarefaction curves for microbial taxonomy and microbial genes annotated by Pfam, rarefaction curves of soil viruses for individual samples (vOTUs and and viral clusters) and their genes (annotated by Pfams) did not reach an asymptote (Extended Data Fig. 1). The number of new and unique vOTUs and viral clusters at the family level (Extended Data Fig. 1b,c) was linearly related to sequencing depth, while viral Pfams displayed slight curvature. These linear relationships were observed when considering 2 TB of metagenomic sequencing––4-fold more sequencing depth than any other soil metagenome in this study and 40-fold more than the JGI recommended sequencing depth for soil samples (45 GB). When considering cumulative unique attributes versus sequencing depth (Extended Data Fig. 2), relationships in vOTUs and viral clusters displayed slight curvature, while viral Pfams neared saturation.

### Microbial hosts of soil viruses

We connected 1,450 viruses to putative hosts of 82 different bacterial and archaeal orders with clustered regularly interspaced short palindromic repeats (CRISPR) spacers. This equates to 2.78% of quality controlled and assured viral contigs that were associated with CRISPR spacer hits, roughly 70% more host assignments than in another recent assessment^4,36^. While we observed a maximum of 73 vOTUs per host (that is, CRISPR spacer), the mean overall vOTU per host ratio was 0.42 (median 0), reflecting the predominance of unique host associations for individual vOTUs.

Out of 1,223 samples with at least one vOTUs assigned to a host, only 72 samples had an average of more than one host sequence per vOTU, underscoring the low abundance of detected hosts across all soils. An average of 0.64 unique host orders were detected per sample, with a maximum ratio of CRISPR spacer hits to viral sequences of 73. Further, samples with a high ratio of vOTU:host almost exclusively were matched to host sequences from a single microbial order, reflecting high phylogenetic conservation of host associations. Of the ten samples with the highest CRISPR spacer sequence to viral sequence ratio, only one contained a CRISPR spacer matching more than one microbial order.

**Fig. 3:**
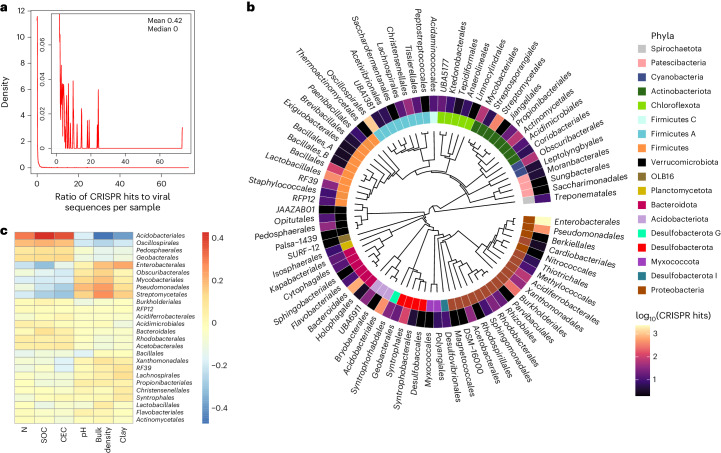
Relationships between soil viruses and their hosts. **a**, A cumulative distribution function plot showing the ratio of CRISPR spacer hits to viral sequences per sample. The inset shows a zoomed portion of the plot from 0 to 0.08 along the *y* axis. **b**, A phylogenetic tree of bacterial hosts at the order level. Phylum level taxonomy is shown in the inner circle, and the abundance of CRISPR spacer hits to each order is shown in the outer circle. Two archaeal orders (*Nitrososphaerales* and *Halobacteriales*) of hosts are not shown. **c**, The correlation between the frequency of CRISPR hits (defined as total CRISPR spacer hits per microbial order) and environmental parameters from SoilGrids250m. Colour denotes Spearman’s rho. Only host orders present in more than five samples are shown in the heatmap.

The most prevalent host taxa were distributed across distantly related phyla, including members of prominent soil orders such as *Pseudomonadales*, *Burkholderiales*, *Acidobacteriales* and *Bacteroidales* (Fig. 3b). The frequency of CRISPR hits associated with *Acidobacteriales*, *Oscillospirales*, *Pedosphaerales* and *Geobacterales* was positively correlated to soil nitrogen, organic carbon and CEC, while *Enterobacterales*, *Obscuribacterales*, *Mycobacteriales*, *Pseudomonadales* and *Streptomycetales* were positively correlated to soil bulk density and, to a lesser extent, pH and clay.

### Metabolic potential encoded by the soil virosphere

**Fig. 4:**
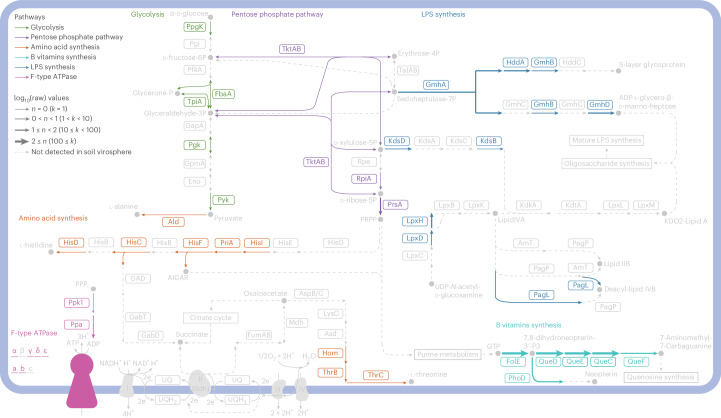
Metabolic potential encoded by the soil virosphere. A cellular diagram depicting portions of the F-type ATPase (map00190), lipopolysaccharide (LPS) biosynthesis pathway (map00540), pentose phosphate pathway (map00030) and vitamin B- and amino acid-related KEGG pathways in the soil virosphere. Genes detected in the soil virosphere are coloured according to pathway type. Undetected genes and associated metabolites (unmeasured) are greyed out. The arrow width denotes gene abundance.

**Extended Data Fig. 3:**
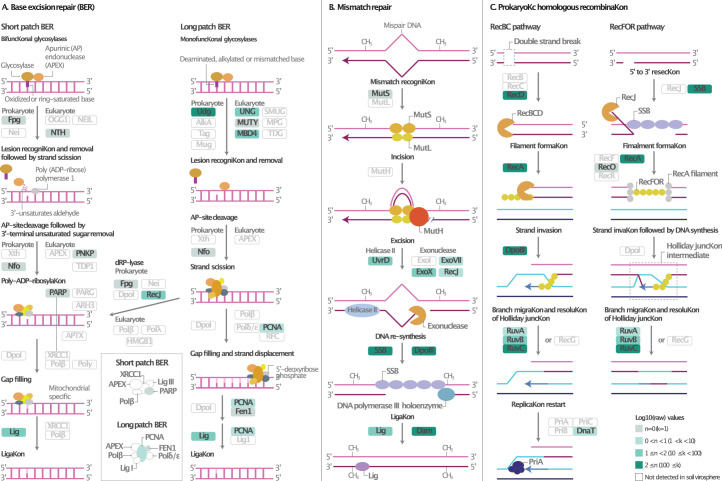
Metabolic potential encoded by the soil virosphere, hallmarks of viral activity. **a**, Base excision repair (map03410), **b**, (prokaryotic) homologous recombination (map03440), and **c**, (prokaryotic) DNA mismatch repair (map03430). KEGG pathways are cropped and/or simplified to enhance visualization. Graphics are adapted from visualizations rendered by Pathview. Color scale denotes the log10 of the total abundance across the entire soil virosphere.

**Extended Data Fig. 4:**
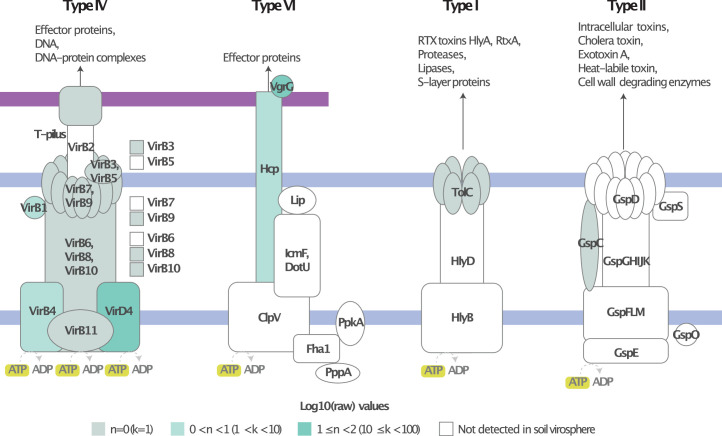
Metabolic potential encoded by the soil virosphere, bacterial secretion systems (map03070). KEGG pathways are cropped and/or simplified to enhance visualization. Graphics are adapted from visualizations rendered by Pathview. Color scale denotes the log10 of the total abundance across the entire soil virosphere.

**Extended Data Fig. 5:**
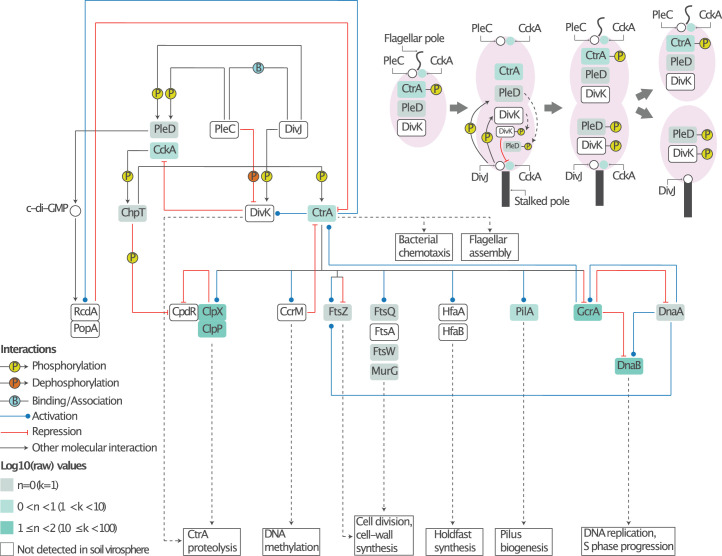
Metabolic potential encoded by the soil virosphere, *Caulobacter* cell cycle (map04112). KEGG pathways are cropped and/or simplified to enhance visualization. Graphics are adapted from visualizations rendered by Pathview. Color scale denotes the log10 of the total abundance across the entire soil virosphere.

**Extended Data Fig. 6:**
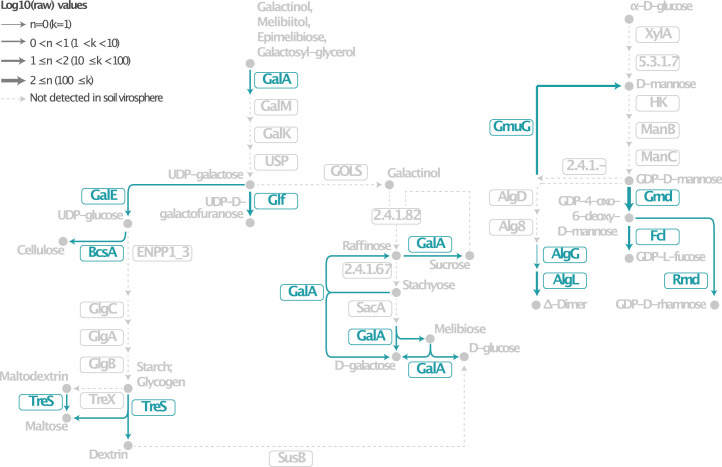
Metabolic potential encoded by the soil virosphere, hallmarks of viral activity. Portions of galactose metabolism (map00052), starch and sucrose metabolism (map00500), and fructose and mannose metabolism (map00051) are depicted. KEGG pathways are cropped and/or simplified to enhance visualization. Graphics are adapted from visualizations rendered by Pathview. Color scale denotes the log10 of the total abundance across the entire soil virosphere.

Because viral gene annotations were sparsely distributed across many functions, we screened all viral genes (regardless of assignment by the AMG pipeline) against KEGG pathways to understand relationships among genes in the context of known metabolic processes. Across the entire soil virosphere, we uncovered portions of KEGG pathways that were mostly complete, including functions involved in amino acid and sugar metabolism and biosynthesis, antibacterial mechanisms, nucleotide synthesis and other viral functions (for example, infection strategies; Extended Data and Fig. 4). For instance, genes associated with DNA mismatch repair (map03430), homologous recombination (map03440) and base excision repair (map03410) were prevalent (Extended Data Fig. 3). Folate biosynthesis was also common in the soil virosphere (map00670 and map00790; Fig. 4). Bacterial secretion systems (map03070; Extended Data Fig. 4), which may be evolutionarily derived from phages^37^, and the *Caulobacter* cell cycle (map04112; Extended Data Fig. 5), which has a distinct division pattern^38^, were rife across soils. The GSV Atlas also contained many viral amino acid biosynthesis/degradation pathways that could be critical in viral life cycles (for example, map00250, map00260, map00270, map00330 and map00340; Extended Data Fig. 6). Finally, we found nearly complete portions of energy-generating pathways including the pentose phosphate pathway and F-type ATPase-mediated portions of photosynthesis. Lipopolysaccharide (LPS) pathway-related genes that may be important as host receptors for bacteriophage and prevention of superinfection were also prevelant^39,40^.

## Discussion

The GSV Atlas demonstrates the immense, unexplored taxonomic and functional diversity of the soil virosphere. Viral diversity in the GSV Atlas appeared to be largely distinct from other global habitats. Nearly 80% of GVS Atlas sequences that clustered with existing sequences in IMG/VR were attributed to soil or soil-like habitats (that is, ‘other terrestrial’ or ‘plant-associated’ (rhizosphere)), underscoring the unique composition of the soil virosphere relative to more well-studied marine and human environments. Additionally, few shared vOTUs and viral clusters between samples may indicate high spatial turnover (that is, changes in soil virosphere composition through space). Recent studies have estimated that soil viral diversity is high, both relative to other viral habitats and relative to soil microbial diversity^7,8,10,22^. However, these estimates have been limited by copious viral and microbial ‘dark matter’ for which no functional or taxonomic assignment is known^14,23,32^. Towards this end, the diversity encompassed by the GSV Atlas can serve as a community resource for characterizing this unknown fraction of the soil virosphere.

Analysis of the GSV Atlas suggests that extreme spatial heterogeneity may be a key feature of the soil virosphere at the global scale. While rapid viral spatial turnover was recently observed across short spatial scales (<10–20 m)^8,10^, there has been no such demonstration of viral biogeography across the world. We propose that high rates of spatial turnover could result from low dispersal rates or distinct temporal dynamics of viral communities relative to other organisms. For example, while dormant microorganisms and relic DNA can persist for months or more^41–43^, the burst of viral cells associated with active infections may generate short-lived pulses of distinct viral communities that do not contribute to relic DNA due to their comparatively small genome sizes versus microorganisms. Additionally, the apparent discrepancies between microbial and viral dispersal processes could be due to the presence of free viruses that are not actively involved in microbial infection^14^, smaller viral genomes that could facilitate physical protection, differences in traits that facilitate dispersal between viruses and microorganisms, variation in bioinformatic pipelines and/or other ecological differences between viruses and microorganisms.

Together, these factors make characterizing the soil virosphere a challenge for the coming decade. When examining individual soil samples, the number of new and unique viral attributes (for example, vOTUs, clusters and Pfams) was linearly related to sequencing depth, suggesting that new viral discoveries are likely to continue with increasingly deep sequencing (Extended Data Fig. 1). This contrasts with rarefaction curves of the soil microbiome and of microbial hosts of soil viruses, which both asymptoted well before sequencing depths of typical soil microbial investigations. Still, when looking at the cumulative number of unique viral attributes detected in all samples collectively (Extended Data Fig. 2), many viral attributes began to saturate with sequencing depth. This suggests that, while individual samples do not capture soil viral diversity, we can begin to constrain the extent of diversity when sequences from thousands of existing samples are aggregated.

Functional diversity encoded by the GSV Atlas revealed the potential for soil viruses to impact biogeochemical cycles, in particular by supporting organic matter decomposition. KEGG pathways and gene annotations represented by the most putative AMGs were related to the metabolism and/or production of sugars common to soils including sucrose, mannose, glucosamine and maltose^44,45^, as well as the decomposition of chitin—one of the most abundant carbon molecules in soil^46^. Our results are consistent with previous work from single locations that have hinted at a wide range of possible soil viral AMGs, including glycoside hydrolases, carbohydrate esterases and carbohydrate-binding modules^15,23,31^. Given that a large proportion of soil microorganisms are infected by viruses at any given time^47^, AMGs encoded by soil viruses have the potential to impact global biogeochemical cycles^15,22,23,31^. The thousands of putative AMGs identified here represent the most extensive survey so far and further impress the importance of the soil virosphere as a reservoir for biogeochemical potential.

Unravelling relationships between viruses and their host communities is imperative to understanding the impact of the virosphere on soil processes. Host presence should be tightly coupled to viral abundance, and in turn, these linkages are mediated by spatial, temporal and environmental factors^15,48,49^. These linkages are also dependent on viral host range (that is, host specificity); higher host specificity should lead to stronger coupling between microbial and viral abundance and community composition. Viral host specificity is also associated with ecological factors that impact microbial community composition and may result in trade-offs between viral growth and the breadth of the host range^11,50–52^. Across the GSV Atlas, there were few hosts per vOTU on average (mean 0.42), and of vOTUs associated with multiple host sequences, the vast majority were linked to multiple hosts of the same phylogenetic clade. While high host specificity has historically been the prevailing paradigm, our work contrasts recent studies suggesting that some soil viruses may have broader host ranges than viruses in other habitats^53,54^.

The ultimate impact of viral predation on soil functions is at least partially associated with the taxonomic distribution of hosts and their variation across soil habitats. Host sequences spanned nearly every major soil microbial clade, consistent with other recent studies (Fig. 3)^22,23,31^. This taxonomic breadth suggests a role for the soil virosphere in most soil habitats. Moreover, some hosts were susceptible to changes in the environment, which may reflect environmental filtering on host communities (which, in turn, determines the amount and type of viruses present) or on viruses directly, which subsequently impacts host community composition^15,27,55,56^. Viral infections have been previously linked to soil parameters including moisture^12,30^ and carbon and nitrogen content^9^. In our analysis, bulk density may serve as a proxy for hydrologic connectivity in the soil matrix. For example, low hydrologic connectivity may create ‘spatial refuges’ for soil bacteria from viral infections^8^, influence the virus–host encounter rates and, thus, structure the soil virosphere and its hosts. Nutrient amendments are also considered to be drivers of the soil virosphere, supporting the relationship we observed between carbon, nitrogen and host taxa.

When examining the functional potential of the soil virosphere, we detected many hallmarks of viral activity––including genes associated with cell lysis, DNA repair/replication and other infection signatures––and viral amino acid biosynthesis/degradation pathways that could be critical in viral life cycles (Fig. 4 and Extended Data Figs. 3–6). The prevalence of viral genes associated with central microbial functions highlights the potential importance of viral activity in soils and the need for targeted approaches to quantify the extent and impact of viral gene expression. For instance, folate and other B vitamins may be logical targets for pathogens as they are key to bacterial growth (map00670 and map00790; Fig. 4)^57,58^. Type IV secretion systems can be used by bacteria to secrete toxins^59^ or as a method for DNA transfer through membranes^60^. The *Caulobacter* cell cycle (map04112; Extended Data Fig. 5) is another promising indicator of viral infections due to its distinct cell division process^38^. Finally, amino acids are building blocks for cellular material and also support soil biogeochemical cycles, as they can enhance carbon cycling through priming effects and/or enhanced nutrient availability^61,62^ (for example, map00500, map00052 and map00051; Fig. 4). Collectively, these pathways demonstrate several possibilities for soil viral impacts on processes that are central to microbial metabolism and biogeochemical cycling of elements in soil.

Beyond these pathways, we highlight three KEGG pathways with near-complete portions represented in the GVS Atlas: F-type ATPase (map00190), pentose phosphate pathway (map00030) and LPS biosynthesis (map00540). Five of seven subcomponents of the F-type ATPase were detected in the soil virosphere, while no V- or A-type ATPases were found. Given the evolutionary similarities between V- and F-type ATPase in particular^63^, the lack of any V- or A-type ATPase components is notable in light of the near-complete F-type ATPase. Though there is some basis for F-type ATPases in viral replication^64^, we also note the possible involvement of F-type ATPase in photosynthetic energy generation^65^. Given the prominence of photosynthetic marine AMGs^26,66^, we highlight the possibility of a viral F-type ATPase as a soil AMG. The pentose phosphate pathway is also a prevalent and important AMG found in marine ecosystems, where viral infection diverts carbon towards the pentose phosphate pathway as an ‘express route’ of energy generation, at the expense of host carbon metabolism (reviewed in ref. ^66^). Finally, we observed nearly complete LPS-related pathways in the GSV Atlas. Phages often carry depolymerases and other enzymes that target LPS or similar outer membrane components to facilitate binding and entry^39^. However, the representation of the LPS biosynthesis pathway by putative soil AMGs indicates that phage may work to change the function of the pathway post-infection, potentially to prevent superinfection^40^. Collectively, we propose that F-type ATPase, pentose phosphate pathway and LPS biosynthesis may be interesting pathways for more targeted investigations into the role of the virosphere in soil microbiome function.

The field of soil viral ecology is poised for rapid expansion, yet several challenges remain in fully characterizing soil viral diversity and function. Overcoming these methodological and ecological hurdles will require broad participation from global researchers. Below, we present a summary of issues, from our perspective, facing the current generation of soil viral ecologists and suggestions for surmounting them.

First, we propose methodological investments to improve viral detection and resolve genomic ‘dark matter’. Metagenomic sequencing can enable the detection of thousands of viruses per soil sample, but the number of viruses detected in soil metagenomes has remained relatively flat over time^4^. In part, this is because soil metagenomic sequences from shotgun sequencing are highly fragmented, leading to lower-quality UViGs^67,68^. Identifying novel viral sequences and assigning viruses to microbial hosts are also limited by the extent of our knowledge of viral diversity; thus, expansion of the known virosphere is needed. Technical advances may improve soil virus identification and host-linkage predictions from shotgun metagenomics, long-read sequencing and/or targeted sequencing approaches. Promising new methods include experimental verification of viral activity^29^, size fractionation (‘viromics’)^7,8,15^, viral isolation^69^, optimized viral nucleic acid extraction^70^, microscopy^29^, combined metagenomic assembly^4^ and long-read and/or single-cell sequencing^71,72^.

Knowledge about soil viral diversity and function is also limited by gaps in field and laboratory experiments. The GSV Atlas demonstrates that extensive, spatially explicit sampling is needed to capture the high spatial turnover of the soil virosphere. The spatial coverage of most ‘global’ ecological studies, including this one, often suffers from large data gaps^73^. Concerted efforts are needed to sample wide spatial domains, including historically undersampled regions, given the high viral diversity uncovered by the GSV Atlas. Expansion of the known virosphere in this way will also help to facilitate tool development. Although we did not assess temporal dynamics, temporally explicit approaches are likewise needed to characterize temporal dynamics in soil viral communities. Further, our functional annotation of viral contigs revealed diverse genes associated with functions relevant to both viral and microbial communities, and it is impossible to know the true functions of viral genes without targeted functional assays. We therefore propose that experiments targeting the expression and auxiliary metabolic function of viral genes are needed to properly assess AMGs in viral communities.

Finally, we still know relatively little about the ecological drivers of soil virus distribution or how to represent these mechanisms in process-based models. Extreme soil virosphere diversity renders some common microbial ecology statistical methods unfeasible, including those often used to test ecological principles (for example, ordinations, distance decay, richness and so on). This highlights the need for innovative statistical approaches to interpret the soil virosphere and to develop new theories surrounding their ecological roles. These advances can help aid development of process-based models, which have made tremendous improvements in representing soil carbon cycles but are missing dynamics involving the soil virosphere.

The GSV Atlas is a new public resource that can help generate hypotheses and provide insight into some of the most pressing challenges in soil viral ecology. We uncovered 616,935 UViGs from global soil samples to show the extreme diversity, spatial turnover and functional potential of the soil virosphere. This includes a wide taxonomic array of microbial hosts of soil viruses, key functions associated with soil carbon cycles and an assortment of viral metabolisms that may be critical to deciphering viral ecological principles in the soil ecosystem. We specifically highlight F-type ATPase, the pentose phosphate pathway and LPS-related genes, as well as enzymes involved in carbohydrate metabolism, as fruitful areas for further investigation. Our work scratches the surface of the soil virosphere and serves as a basis for tool, theory and model development to further advance soil ecology, biogeochemistry, ecology and evolution.

## Methods

### Data collection and curation

We collected a total of 2,953 soil metagenomic samples from major repositories and ecological networks including the JGI IMG/M platform, MG-RAST metagenomics analysis server, Global Urban Soil Ecological Education Network, Earth Microbiome Project and National Ecological Observatory Network plus submissions from individual collaborators. This included 1,552 samples not previously included in IMG/M (Figs. 1 and 2). All dataset authors were contacted for data re-use permissions.

For samples collected via JGI IMG/M, we retrieved all studies with GOLD^74^ ecosystem type of ‘Soil’ as of August 2020. We manually curated metagenomic sequences to remove misclassified data as follows. We removed samples from studies with the following: (1) GOLD ecosystem types: rock-dwelling, deep subsurface, plant litter, geologic, oil reservoir, volcanic and contaminated; (2) GOLD ecosystem subtypes: wetlands, aquifer, tar, sediment, fracking water and soil crust; (4) words in title: wetland, sediment, acid mine, cave wall surface, mine tailings, rock biofilm, beach sand, petroleum, stalagmite, subsurface hydrocarbon microbial communities, vadose zone, mud volcano, fumarolic, enriched, composted filter cake, ice psychrophilic, oil sands, groundwater, contaminated, rock biofilm, deep mine, coal mine fire, hydrocarbon resource environments, marine, enrichment, groundwater, mangrove, saline desert, hydroxyproline, rifle, coastal, compost, biocrust, crust, creosote, soil warming, testing DNA extraction and/or agave; (5) GOLD geographic location of wetland; and (6) GOLD project type of Metagenome - Cell Enrichment. Additionally, sample names that indicated experimental manipulation (for example, CO_2_ enrichment or nitrogen fertilization) or were located in permafrost layers were manually excluded. This resulted in 1,480 curated metagenomes from publicly available data in IMG/M.

After collating samples from JGI IMG/M and the newly collected samples from external networks and collaborators, the final dataset consisted of 2,953 soils with 2,015,688,128 contigs, representing 1.2 terabases of assembled DNA sequences.

In parallel, we retrieved mean values for soil parameters from the SoilGrids250m database from 0–5 cm (ref. ^33^). SoilGrids250m is a spatial interpolation of global soil parameters using ~150,000 soil samples and 158 remote sensing-based products. Here, we focus on six parameters often associated with soil microbial communities: bulk density, CEC, nitrogen, pH, SOC and clay content. Because we focused on spatial dynamics and soils were collected at various times, we did not include temporally dynamic variables such as soil moisture or temperature in our set of environmental parameters, though we acknowledge they may have profound impacts on the soil virosphere.

### Assembly and annotation of samples added to IMG/M

To standardize data analysis across all samples, the 1,552 soil metagenomic samples not collected from IMG/M were analysed using the JGI’s Metagenome Workflow^75^. In brief, samples were individually assembled using MetaSpades v3.1. A total of 1,476 of the 1,552 assembled soil samples passed default quality control thresholds^76^, yielding 133 gigabases of assembled DNA in 241,465,924 contigs. Additionally, three very large metagenomes (>1 TB each) were assembled separately due to computational limitations in standard workflows^77^. The resulting assemblies were assigned GOLD identification numbers and imported into IMG/M and processed using IMG/M Metagenome Annotation Pipeline v5.0.0 to align with data obtained directly from IMG/M^75^.

### Virus identification, clustering, and host prediction

We performed an initial identification of viral contigs using a modified version of the IMG/VR v3’s virus identification pipeline (code available at ref. ^78^)^35,36^. The pipeline identifies viruses on the basis of the presence of 23,841 virus protein families, 16,260 protein families of microbial origin from the Pfam database^79^ and VirFinder^80^ to identify putative viral genomes in contigs that were at least 1 kb long. During the course of this study, geNomad v1.3.3 (ref. ^81^), a tool for virus identification with improved classification performance was released and incorporated into our pipeline to improve prediction confidence and perform taxonomic assignment. We further processed predicted viral sequences using CheckVv1.0.1 (database version 1.5)^82^ to assess the quality of the viral genomes. As this study focused on non-integrated virus genomes, contigs that were flagged by either geNomad or CheckV as proviral were discarded. From the remaining contigs, virus genomes were selected using the following rules: (1) contigs of at least 1 kb with high similarity to genomes in the CheckV database (that is, that had high- or medium-quality completeness estimates) or that contained direct terminal repeats were automatically selected; (2) contigs longer than 10 kb were required to have a geNomad virus score higher than 0.8 and to either encode one virus hallmark (for example, terminase, capsid proteins, portal protein and so on), as determined by geNomad, or to have a geNomad virus marker of at least 5.0; (3) contigs shorter than 10 kb and longer than 5 kb were required to have a geNomad virus score higher than 0.9, to encode at least one virus hallmark and to have a virus marker enrichment higher than 2.0. This resulted in 49,649 viral contigs that we used for downstream analysis. All viral contigs are available at ref. ^83^.

Viral genomes were clustered into vOTUs following MIUViG guidelines (95% average nucleotide identity, 85% aligned fraction^34^). In brief, we performed an all-versus-all BLAST (v2.13.0+, ‘-task megablast -evalue 1e-5 -max_target_seqs 20000’) search to estimate pairwise average nucleotide identities and aligned fractions (AFs), as described in Nayfach et al.^82^ and employed pyLeiden (available at ref. ^84^) to cluster genomes, using as input a graph where pairs of genomes that satisfied the MIUViG criteria were connected by edges. Viruses were also grouped at approximate genus level (40% average amino acid identity, 20% shared genes) and family level (20% average amino acid identity, 10% shared genes) clusters using DIAMOND^85^ for protein alignment and Markov Cluster Process^86^ for clustering^35^.

Viral sequences were assigned to putative host (bacterial and archaeal) taxa through matches to a previously described database of CRISPR spacers of 1.6 million bacterial and archaeal genomes from NCBI GenBank and MAGs (release 242; 15 February 2021)^87–91^. Sequences of viral genomes were queried against the spacer database^92^ using blastn (v2.9.0+, parameters: ‘-max_target_seqs = 1000 -word_size = 8 -dust = no’). Only alignments with at least 25 bp and fewer than two mismatches, and that covered ≥95% of the spacer length, were considered. Viral sequences were assigned to the host taxon at the lowest taxonomic rank that had at least two spacers matched and that represented >70% of all matches.

### Potential AMG prediction

We leveraged an intermediate output of geNomad (v1.3.3)^81^ (‘genes.tsv’) to screen putative AMGs on the detected viral contigs. Proteins of the viral contigs were annotated by virus- and host-specific markers implemented in geNomad. The identified viral hallmark (for example, terminase and major capsid protein) and non-hallmark proteins were labelled as ‘VV-1’ and ‘V*-0’ in geNomad output, respectively. The rest of the viral proteins of the detected viral contigs that were annotated as non-virus-specific or unclassified were then classified into five categories of putative AMGs based on the presence of viral hallmark or non-hallmarks up- or downstream as mentioned previously^30^. The AMGs with both virus-specific genes (‘VV-1’ or ‘V*-0’) were retained for the following analysis. To improve the functional annotations of the putative AMGs and highlight the viral potentials of metabolizing carbohydrates and glycoconjugates, the AMG proteins were also annotated by Carbohydrate-Active enZYmes (CAZy) Database and KEGG database using the default settings in addition to the functional annotation databases implemented in geNomad. The putative AMG was assigned to the functional annotation with the highest bitscore (for example, duplicate annotations were not allowed). Following Hurwitz and U’Ren^66^ and Hurwitz et al.^93^, we further screened putative AMGs to remove genes not found in KEGG pathways. Additionally, in recognition of the ambiguity in distinguishing genes encoding auxiliary metabolic functions versus core metabolic processes^66^, we discuss the resulting set of genes presented here as ‘putative AMGs’.

### Statistical analysis

All statistical analyses and data visualizations were performed using *R* v4.1.0 (ref. ^94^). We used the following packages for data manipulation and visualization: ggplot2 (ref. ^95^), reshape2 (ref. ^96^), pheatmap^97^, Hmisc^98^, ggpubr^99^, RColorBrewer^100^, maps^101^, stats geosphere^102^, plyr^103^, dplyr^104^ and stringr^105^. Additional packages pertaining to specific analyses are listed below.

We generated rarefaction curves for individual samples and for cumulative sequencing depth (Extended Data Figs. 1 and 2) using the ‘phyloseq’ package^106^ and custom R plots, respectively. Samples containing fewer than five vOTUs, viral clusters or viral Pfams; or fewer than 100 CRISPR-spacer-based host taxa, microbial Pfams or microbial taxa were removed for visual clarity. Removed samples followed the same general trends as shown in Extended Data Fig. 1. To visualize saturation across cumulative sequencing depth (Extended Data Fig. 2), we ordered samples from lowest to highest total assembled base pairs and progressively added them along the *x* axis. On the *y* axis, we plot the associated cumulative number of unique attributes.

A phylogenetic tree of CRISPR-spaced-based host taxa was generated at the order-level using phyloT v2 (https://phylot.biobyte.de/), an online tree generator based on the Genome Taxonomy Database. Then, we visualized the tree in R using the packages ‘ggtree’^107^, ‘treeio’^108^ and ‘ggnewscale’^109^. To examine relationships between common microbial hosts of soil viruses and soil properties, we first downloaded data describing bulk density, CEC, nitrogen, pH, SOC and clay content from the SoilGrids250m database^33^ using the ‘soilDB’ package^110^. Mean values of soil properties from 0 to 5 cm were correlated to the total number of CRISPR spacer hits per microbial order using Spearman correlation.

Finally, we mapped genes detected across the entire soil virosphere (that is, all samples combined) to their corresponding KEGG pathways using the ‘pathview’ package in R^111^. Gene abundances were converted by log base 10 for visualization.

### Reporting summary

Further information on research design is available in the Nature Portfolio Reporting Summary linked to this article.

### Supplementary information

Reporting Summary

## Data Availability

The GSV Atlas is available for download at 10.25584/2229733 (ref. ^83^). It includes all UViGs regardless of quality (File 1, 616,935 UViGs), data associated with each contig that passed QA/QC (File 2, 49,649 contigs), predicted viral protein sequences (File 3, 402,882 predicted protein sequences), data associated with each gene (File 4, 1,432,147 genes), geographic and physico-chemical data of the curated soil samples (File 5, 2,953 samples) and a readme file (File 6).

## Code availability

Code for sequence processing is described in Nayafch et al.^35^ and is available in the materials associated with those publications. Github repositories associated with this publication are available at https://github.com/snayfach/MGV/tree/master/viral_detection_pipeline (ref. ^78^), https://github.com/apcamargo/pyleiden (ref. ^84^) and https://github.com/apcamargo/genomad (ref. ^81^).
